# Characterization of Newly Isolated *Rosenblumvirus* Phage Infecting *Staphylococcus aureus* from Different Sources

**DOI:** 10.3390/microorganisms13030664

**Published:** 2025-03-15

**Authors:** Paloma Cavalcante Cunha, Pedro Samuel de Souza, Ana Julia Dill Rosseto, Isabella Ribeiro Rodrigues, Roberto Sousa Dias, Vinícius da Silva Duarte, Davide Porcellato, Cynthia Canêdo da Silva, Sérgio Oliveira de Paula

**Affiliations:** 1 Department of Microbiology, Federal University of Viçosa, Avenida Peter Henry Rolfs, s/n, Viçosa 36570-900, Minas Gerais, Brazil; paloma.cavalcante@ufv.br (P.C.C.); ccanedosilva@gmail.com (C.C.d.S.); 2 Department of General Biology, Federal University of Viçosa, Avenida Peter Henry Rolfs, s/n, Viçosa 36570-900, Minas Gerais, Brazil; samuelhomero59@gmail.com (P.S.d.S.); ana.rosseto@ufv.br (A.J.D.R.); isabella.r.rodrigues@ufv.br (I.R.R.); rosousa318@gmail.com (R.S.D.); 3 Faculty of Chemistry, Biotechnology and Food Science, The Norwegian University of Life Sciences, P.O. Box 5003, 1432 Ås, Norway; vinicius.da.silva.duarte@nmbu.no (V.d.S.D.); davide.porcellato@nmbu.no (D.P.)

**Keywords:** *Staphylococcus* phage, *Rosenblumvirus*, biocontrol, host range

## Abstract

*Staphylococcus aureus* is a globally significant pathogen associated with severe infections, foodborne illnesses, and animal diseases. Its control has become increasingly challenging due to the spread of antibiotic-resistant strains, highlighting the urgent need for effective alternatives. In this context, bacteriophages have emerged as promising biocontrol agents. This study aimed to characterize the newly isolated *Staphylococcus* phage CapO46 and evaluate its efficacy in reducing *S. aureus* in milk. Identified as a new species within the *Rosenblumvirus* genus, CapO46 exhibited a podovirus-like structure and a small linear dsDNA genome (17,107 bp), with no lysogeny-related, antimicrobial resistance, or virulence genes. Host range assays demonstrated its ability to infect all 31 *S. aureus* isolates from two different countries and in diverse environmental contexts, achieving high efficiency of plating (EOP > 0.5) in 64.5% of cases. Kinetic analyses revealed rapid adsorption and a short latent period, with a burst size of approximately 30 PFU/cell. In UHT whole-fat milk, CapO46 achieved a maximum reduction of 7.2 log10 CFU/mL in bacterial load after 12 h, maintaining significant suppression (1.6 log10 CFU/mL) after 48 h. Due to its genetic safety, high infectivity across multiple isolates, and antimicrobial activity in milk, CapO46 can be considered a promising candidate for *S. aureus* biocontrol applications.

## 1. Introduction

*Staphylococcus aureus* is a bacterium of great global significance, with a substantial impact on public health, food safety, and animal health. This pathogen is responsible for a wide range of infections in humans, ranging from mild conditions, such as skin infections, to severe and potentially fatal diseases, such as pneumonia, sepsis, and endocarditis [1]. In food, *S. aureus* is one of the leading causes of foodborne intoxication due to its production of heat-stable enterotoxins [2,3]. In animal health, it stands out as the main causative agent of bovine mastitis, a disease that compromises milk quality, reduces productivity, and causes significant economic losses in the dairy production chain [4,5]. The control of *S. aureus* is challenging due to its ability to form biofilms, which protect bacterial cells from the immune system and antimicrobials, as well as its increasing resistance to conventional antibiotics, such as beta-lactams, which has given rise to the well-known methicillin-resistant strains (MRSA—methicillin-resistant *Staphylococcus aureus*) [1,2]. These features underscore the urgent need to develop alternative strategies for controlling this pathogen in diverse settings.

Bacteriophages (phages) have emerged as promising tools for bacterial biocontrol due to their high specificity, lytic efficacy, and environmental safety [6,7,8]. These viruses selectively target and destroy bacteria, offering a significant advantage over antibiotics. Additionally, phages can degrade biofilms using specific enzymes such as depolymerizes and endolysins, enhancing their effectiveness in environments where biofilms protect bacteria or contribute to resistance [9,10]. In the agro-food industry, phages have been used as food biopreservatives, biopesticides to mitigate disease loads in crops, feed additives to manage bacterial infections in animals, and surface sanitizers in livestock facilities and food processing industries [11,12]. Phage application can reduce pathogens without harming beneficial microorganisms thus improving animal health, preserving product quality, and ensuring consumer safety [13,14,15]. Moreover, introducing phages as a natural alternative could promote more sustainable agricultural practices by reducing antibiotic use and minimizing pharmaceutical residues in the environment.

For safe and effective use in commercial agro-food applications, phages must exhibit several desirable characteristics [12]. These include the absence of genes associated with antibiotic resistance or virulence, as well as the absence of lysogeny-related genes (i.e., they must be strictly lytic). Additionally, they should be highly specific to the target bacterium to prevent disruptions to the beneficial microbiota, and they must be stable to maintain efficacy under varying environmental conditions, such as temperature and pH fluctuations. Moreover, phages should be easily producible on a large scale to ensure commercial viability. Thus, the use of phages requires not only their isolation but also their biological and genomic characterization to enable a full exploration of their practical applications [16].

In this study, we report the isolation of the phage CapO46, obtained from effluents of a goat farm in Brazil, and evaluate its host range in *S. aureus* isolates from two different countries and diverse environmental contexts. Through genomic analyses, we determine the taxonomic classification of this novel phage and its genetic safety. Finally, we investigate the antimicrobial efficacy of CapO46 in *S. aureus*-contaminated ultra-high-temperature (UHT) whole-fat milk, highlighting its potential as a biocontrol agent under practical conditions.

## 2. Materials and Methods

### 2.1. Bacterial Strains and Growth Conditions

The *S. aureus* O46, isolated from an ewe with mild mastitis [17,18], was used as the host for phage isolation and propagation. For the host range investigation, *S. aureus* isolates from animals, humans, and cow milk were selected from the various bacterial libraries of collaborating research laboratories (Supplementary Table S1). Some isolates, including those kindly provided by EMBRAPA Gado de Leite (Juiz de Fora, MG, Brazil), had been previously characterized [19,20,21,22,23]. The Norwegian isolates were obtained from the SciFood Lab at the Norwegian University of Life Sciences (NMBU), Norwegian city, Norway, and there was no information regarding genetic variability. The *S. aureus* strain NCTC 8325-4 was kindly provided by Professor Morten Kjos from NMBU.

Isolates were routinely cultured in Brain Heart Infusion (BHI) broth (Kasvi, São Paulo, SP, Brazil) and BHI agar (1.5% agar *w*/*v*), then incubated at 37 °C. Optical density was measured using a spectrophotometer (Shanghai Spectrum SP-1105, Shanghai, China) at 600 nm (OD_600_) to determine the phase of bacterial growth.

### 2.2. Phage Isolation and Propagation

For phage isolation, a sample was collected from the goat farm wastewater at UFV, processed, and then enriched following the method of Van Twest and Kropinski [24]. Briefly, the sample was centrifuged at 10,000× *g* for 15 min at 4 °C, and the supernatant was filtered through a 0.45 µm PES membrane (Millipore, Billerica, MA, USA). Next, 10 mL of sterile double-strength BHI broth was inoculated with 0.1 mL of *S. aureus* O46 culture (Table S1) in the logarithmic growth phase (OD_600_ = 0.4) and mixed with 10 mL of the filtered wastewater sample. The mixture was incubated at 37 °C with shaking at 100 rpm for 24 h. Following incubation, the mixture was centrifuged at 10,000× *g* for 10 min at 4 °C, and the supernatant (lysate) was filtered through a 0.22 µm PES membrane (Millipore, Billerica, MA, USA). The lysate was then diluted in SM buffer (5.8 g/L NaCl, 2.0 g/L MgSO_4_·7H_2_O, 50.0 mL/L Tris-HCl 1M, 5.0 mL/L 2% *w*/*v* gelatin, pH 7.5) and plated using the double-layer agar (DLA) technique [24,25]. The plates were incubated overnight at 37 °C. Among the resulting lysis plaques, one was selected, excised, and replated. This process was repeated at least three times to ensure the isolation of a single phage. Finally, the phage was propagated in BHI broth as described by Sambrook et al. [26], filtered through a 0.22 µm PES membrane, titrated, and stored at 4 °C.

### 2.3. Transmission Electron Microscopy

For the viral morphology analysis, transmission electron microscopy (TEM) was performed. Briefly, 5 µL of a high-titer viral suspension (>10^10^ PFU/mL) was applied to Formvar^®^-coated grids with 200-mesh support for 5 min, followed by negative staining with 5 µL of 3% (*w*/*v*) uranyl acetate for 15 s. The grids were then dried in a vacuum chamber for approximately 24 h and analyzed using a Zeiss EM 109 transmission electron microscope (Zeiss, Oberkochen, Germany) at the Center for Microscopy and Microanalysis (UFV). The acquired images were analyzed for capsid and tail dimensions using the ImageJ v1.54g software (National Institutes of Health, Bethesda, MD, USA). Measurements were obtained from three separate images to ensure accuracy and consistency.

### 2.4. Host Range

The ability of the phage to infect different bacterial strains was initially assessed by spotting 10 µL of the phage suspension onto the surface of a double-layer agar plate inoculated with the test bacterium (Table S1). The plates were incubated overnight at 37 °C. Bacteria that exhibited clear spots or plaques at the inoculation site were recorded as sensitive to the phage and subsequently used in the efficiency of plating (EOP) assay to evaluate productive infection [27]. For the EOP assay, the phage lysate was serially diluted in SM buffer and spot-plated onto double-layer agar containing the sensitive bacteria. The plates were incubated overnight at 37 °C, and the PFU/mL was calculated the following day. All experiments were performed in triplicate. To determine the EOP, the mean PFU/mL of the test bacterium was divided by the mean PFU/mL of the original host bacterium. The EOP for a specific phage–bacterium combination was classified as “high production” when the ratio was 0.5 or higher, meaning that productive infection in the target bacterium resulted in at least 50% of the PFU/mL observed in the original host. It was considered “medium production” when the EOP ranged from 0.1 to less than 0.5, “low production” when it ranged from 0.001 to less than 0.1, and “inefficient” when it was greater than 0 but less than or equal to 0.001. If no lysis plaques were observed, the EOP was classified as “no production”.

### 2.5. Adsorption and One-Step Growth Curve

To evaluate the kinetics of viral infection, adsorption and one-step growth curve assays were performed in triplicate. For both assays, the isolate *S. aureus* O46 was grown in a BHI medium until reaching the mid-exponential phase (OD_600_ = 0.4).

The adsorption assay was conducted as described by Hyman and Abedon [28] but with modifications. The bacterial culture was infected with the phage at a multiplicity of infection (MOI) of 0.01 and incubated at 37 °C for 14 min. Samples were collected immediately and at 2 min intervals, post-infection, then centrifuged at 13,000× *g* for 1 min. The supernatants were diluted and plated using the double-layer agar (DLA) method to determine the titers of non-adsorbed phages. The adsorption curve was expressed as relative adsorption (%), calculated as follows:

100 × [1 − (titer of non-adsorbed phages at each time point/initial titer of phages)].(1)

The one-step growth curve assay was performed as reported by Sharifi et al. [29] but with some modifications. Briefly, 10 mL of bacterial culture was mixed with 100 µL of phage suspension to achieve an MOI of 0.01. The mixture was incubated for 10 min at 37 °C to allow for viral adsorption, then centrifuged (10,000× *g*, 5 min) to remove non-adsorbed phages. The supernatant was discarded, and the pellet was resuspended in 10 mL of pre-warmed BHI broth and incubated again at 37 °C with shaking at 100 rpm. Samples were collected immediately after resuspension (T0) and at 5 min intervals for 60 min and then plated using the DLA method. The latent period was defined as the time interval between adsorption and the onset of the first burst, as indicated by the initial rise in phage titer. The burst size was calculated by dividing the mean titer observed after the first burst by the average initial titer before the burst.

### 2.6. Genome Sequencing and Bioinformatics Analysis

For phage genome extraction, the phage lysate was first treated with 5 µL/mL of DNase I (Thermo Fisher Scientific, Waltham, MA, USA) at 37 °C for 60 min to eliminate free host DNA contamination. DNase I was inactivated with 50 mM EDTA by incubation at 65 °C for 10 min, and the viral DNA was subsequently extracted using the mag™ Midi DNA Purification Kit (LGC Biosearch Technologies, Teddington, UK), following the manufacturer’s instructions. The sequencing library was prepared using 1 ng of input DNA with the Illumina Nextera XT DNA Kit (Illumina, San Diego, CA, EUA), and normalization was performed using the SequalPrep™ Normalization Kit (Thermo Fisher Scientific). Whole-genome sequencing was conducted by Novogene (Cambridge, UK) using the Illumina NovaSeq X Plus 2 × 150 bp paired-end.

Raw reads were quality-filtered and assembled into contigs and scaffolds using SPAdes v3.13.0 through the Bacterial and Viral Bioinformatics Resource Center (BV-BRC) (https://www.bv-brc.org/, accessed on 7 May 2024), with read trimming (TrimGalore) and normalization enabled in the ‘True’ mode. The minimum contig length and coverage thresholds were set to 1000 and 5, respectively. The obtained CapO46 genome sequence was analyzed using the BLASTn tool in the NCBI database (https://blast.ncbi.nlm.nih.gov/Blast.cgi, accessed on 7 May 2024) to identify the closest related sequences and their corresponding subfamily. All RefSeq genomes of the members of the identified subfamily were retrieved from the NCBI Virus database (https://www.ncbi.nlm.nih.gov/labs/virus/vssi/#/, accessed on 7 May 2024). The CapO46 genome was then incorporated into the subfamily genome dataset and analyzed using the Viral Proteomic Tree (ViPTree) server [30] (https://www.genome.jp/viptree/, accessed on 7 May 2024) to determine the phylogenetic relationships and generate a proteomic tree. Intergenomic comparisons were performed via the VIRIDIC web server [31] (http://rhea.icbm.uni-oldenburg.de/viridic/, accessed on 7 May 2024) following the International Committee on Taxonomy of Viruses (ICTV) guidelines, which define species and genus boundaries at similarity thresholds of 95% and 70%, respectively [32].

Gene prediction for the CapO46 genome was performed using Prokka v1.14.6+galaxy1 on the Phage Galaxy web platform (https://phage.usegalaxy.eu/, accessed on 7 January 2025). Automatic annotations were manually curated using the BLASTp tool in NCBI and the InterProScan web service (https://www.ebi.ac.uk/interpro/, accessed on 7 January 2025) to generate the final consensus annotation table. The phage genomic map was constructed using the Proksee platform [33] (https://proksee.ca/, accessed on 7 January 2025). Antimicrobial resistance genes and virulence factors were identified using ResFinder 4.5.0 [34] and VirulenceFinder 2.0.5 [35,36] on the CGE platform (https://www.genomicepidemiology.org/, accessed on 7 January 2025).

The complete CapO46 genome sequence has been deposited in the NCBI GenBank under accession number PV007823.

### 2.7. Phage Bactericidal Activity in UHT Whole-Fat Milk

The bactericidal activity of the phage in UHT whole-fat milk (3% fat) was evaluated as described by García et al. [37], with modifications. The bacterial culture was grown to the mid-exponential phase (OD_600_ = 0.4) and subsequently diluted in phosphate-buffered saline (PBS). Aliquots of 50 µL of the diluted bacterial culture and 50 µL of the phage suspension, also diluted in PBS, were added to tubes containing 5 mL of UHT whole-fat milk to achieve a multiplicity of infection (MOI) of 1000. Positive control tubes, inoculated with bacteria only, received 50 µL of sterile PBS instead of phage. Negative control tubes, containing only sterile PBS and without bacterial or phage inoculation, were used to check for milk contamination. The tubes were incubated at 37 °C, and aliquots were collected at 0, 3, 6, 12, 24, and 48 h. The aliquots were diluted in PBS, and the bacterial concentrations (CFU/mL) were determined using the spread-plate method. The phage concentrations (PFU/mL) were determined by the double-layer agar (DLA) method. The entire experiment was conducted in triplicate.

### 2.8. Statistical Analysis

The statistical significance of the means was assessed using GraphPad Prism software v 8.3.0 (538). First, the normality of the data was evaluated. If the data were normally distributed, an independent t-test was used to compare the means of the control and CapO46-treated groups at each time point (0, 3, 6, 12, 24, and 48 h). If the data were not normally distributed, the Mann–Whitney test was applied. Significant differences were considered for *p*-values < 0.05.

## 3. Results

### 3.1. Isolation and Morphology of CapO46 Phage

**Figure 1:**
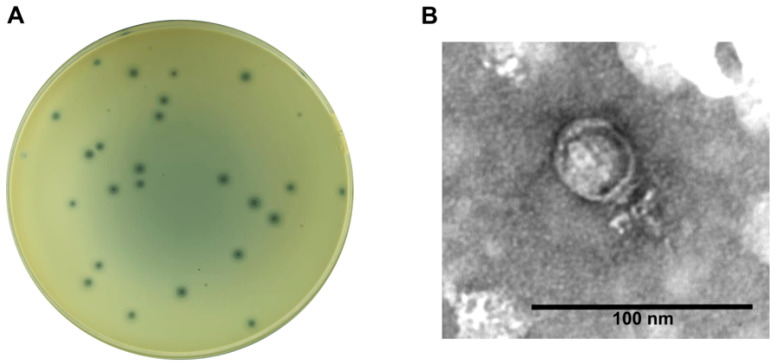
CapO46 phage (**A**) Plaque morphology on BHI medium and (**B**) Transmission electron micrograph of negatively stained phage particle.

The lytic *Staphylococcus* phage CapO46, hereafter referred to as CapO46, was isolated from goat farm (*caprinocultura* in Brazilian Portuguese) wastewater using an *S. aureus* strain (O46) obtained from an ewe with mild mastitis [17,18]. The phage readily achieves high titers (>10^10^ PFU/mL) when routinely propagated in its isolation host. In the BHI medium, the phage produces clear, round lysis plaques approximately 1 mm in diameter, surrounded by a translucent halo of about 3 mm in diameter (Figure 1A). Electron micrographs reveal that the phage has a podovirus morphology, with an icosahedral head approximately 40 nm in diameter (*n* = 3) and a short, non-contractile tail about 23 nm in length (Figure 1B).

### 3.2. CapO46 Genome Annotation and Phylogenetic Analysis

**Figure 2:**
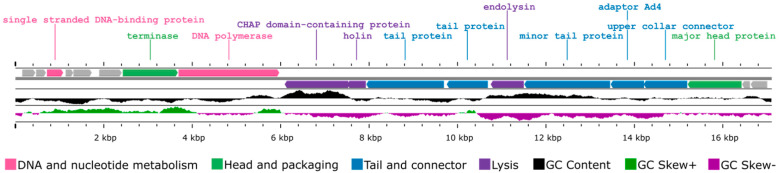
Genome map of phage CapO46 generated by Proksee based on ORF annotations from Prokka. The genes are color-coded based on their predicted functionality, and the GC content is represented by different colors using CGView v1.0.2.

CapO46 is a virulent phage with a small dsDNA genome of 17,107 bp and a G + C content of 29.08%. Of the 19 putative open reading frames (ORFs) identified, 12 were predicted to be functional, and 7 were annotated as hypothetical proteins of unknown function (Figure 2). The functional ORFs were categorized into the following four modules: DNA and nucleotide metabolism (two ORFs), head and packaging (two ORFs), tail and connector (four ORFs), and lysis (three ORFs). No genes encoding tRNA, virulence factors, or antibiotic resistance genes were identified in the phage genome.

**Figure 3:**
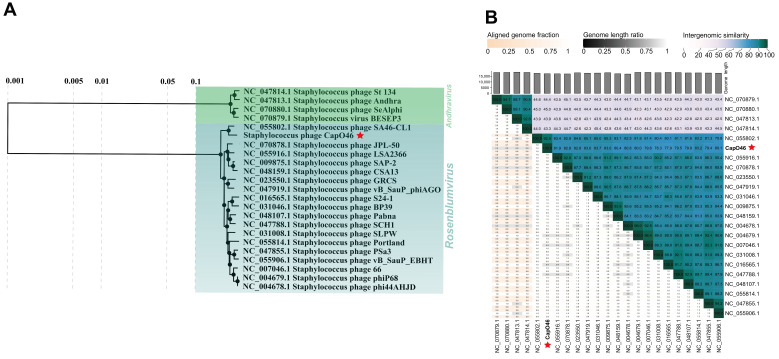
CapO46 phage (**A**) Phylogenetic tree and (**B**) Intergenomic similarity scores, showing its relationship with RefSeq genomes of members of the *Rakietenvirinae* subfamily. The CapO46 phage is highlighted with a red star in both images.

Regarding the taxonomic analyses, CapO46 was grouped in the same clade of the proteomic tree as representatives of the *Rosenblumvirus* genus (*Rakietenvirinae* subfamily, *Rountreeviridae* family), alongside a clade formed by members of the same subfamily (*Andhravirus* genus) (Figure 3A). In the VIRIDIC analysis (Figure 3B), the highest similarity score obtained was with *Staphylococcus* phage SA46-CL1 (RefSeq accession: NC_055802.1, similarity score: 92.9), indicating that CapO46 represents a new species within the *Rosenblumvirus* genus, according to ICTV criteria.

### 3.3. CapO46 Host Range

The CapO46 phage exhibited a wide strain specificity, being capable of infecting various *S. aureus* isolates from both Brazil and Norway, additionally of its Franch isolation strain. All 31 *S. aureus* isolates tested were lysed to some extent by the phage (Table 1). The EOP assays revealed that the phage was able to infect 64.5% of the isolates with high EOP (20/31), 9.7% with medium EOP (3/31), and 25.8% with inefficient EOP (8/31). Notably, the majority of the isolates classified as having high EOP (14/20) were Norwegian *S. aureus* strains. The phage did not infect any strain of non-aureus staphylococci.

### 3.4. CapO46 Adsorption and One-Step Growth Curve

**Figure 4:**
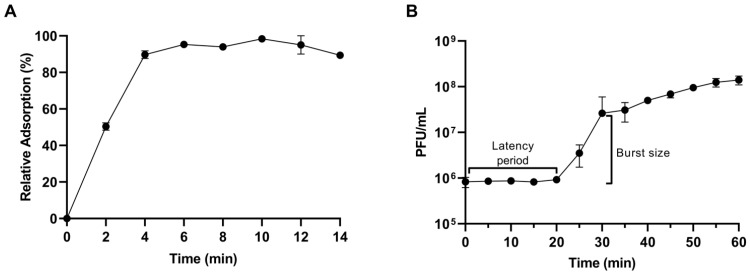
(**A**) Relative adsorption and (**B**) One-step growth curve of CapO46 phage at an MOI of 0.01.

The adsorption of CapO46 phage occurs rapidly, with approximately 50% (50.3% ± 1.5%) of the phage particles adsorbed to bacterial cells in just 2 min and nearly 100% (98.3% ± 1.1%) adsorbed within 10 min (Figure 4A). The one-step growth curve shows that the phage’s latent period at an MOI of 0.01 is 20 min, and the burst size, which occurs at 30 min, is on average 30 (30.7 ± 3.8) PFU/cell (Figure 4B).

### 3.5. Capo46 Bactericidal Activity in UHT Whole-Fat Milk

**Figure 5:**
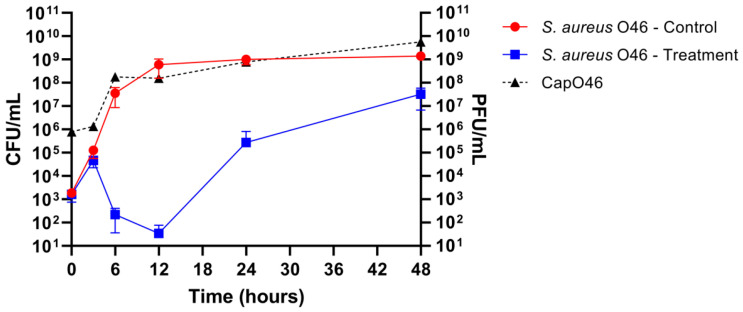
Bactericidal activity of phage CapO46 against *S. aureus* O46 in UHT whole-fat milk over 48 h. The graph illustrates the bacterial load (*S. aureus* O46 CFU/mL) in untreated samples (red line, “*S. aureus* O46—Control”) and the reduction in bacterial counts following phage treatment (blue line, “*S. aureus* O46—Treatment”). Phage replication is represented by titers in PFU/mL (dotted black line, “CapO46”).

The potential of phage CapO46 to inhibit *S. aureus* growth in UHT whole-fat milk was evaluated over 48 h at 37 °C (Figure 5). Treatment with the phage at an MOI of 1000 demonstrated a significant reduction (*p*-value < 0.05) in bacterial load, starting at 6 h of incubation. By 6 h, the bacterial counts had decreased by 5.2 log10 CFU/mL, reaching a peak reduction of 7.2 log10 CFU/mL at 12 h. Although the bacterial population began to regrow after 24 h of incubation, its concentration remained lower in the phage-treated samples compared to the control, with bacterial loads reduced by 3.5 log10 CFU/mL at 24 h and 1.6 log10 CFU/mL at 48 h relative to the untreated control.

The phage titers were also assessed at different time points, demonstrating the effective replication of phage CapO46 in the milk (Figure 5). After 3 h of incubation, a slight increase of 0.23 log10 PFU/mL was observed, indicating limited initial activity. However, a substantial increase was recorded at 6 h, with the mean phage titer reaching 8.13 log10 PFU/mL, representing a 2.08 log10 increase compared to the initial time. Between 6 and 12 h, the phage titers remained stable, with a mean value of 8.12 log10 PFU/mL at 12 h. A further increase was observed at 24 h, with a mean titer of 8.65 log10 PFU/mL, corresponding to a 0.53 log10 PFU/mL rise from 12 h. At the final time point (48 h), the mean phage titer reached 9.60 log10 PFU/mL, representing the highest titer observed and an overall increase of 3.78 log10 PFU/mL from the initial time.

## 4. Discussion

The findings of this study highlight the potential of the phage CapO46 as a promising candidate for *S. aureus* biocontrol applications. The successful isolation of CapO46 from farm wastewater underscores the effectiveness of bacterium-enrichment methodologies for recovering lytic phages and emphasizes the importance of such environments as sources for discovering novel viruses [24,38]. Morphological characterization revealed a podovirus-like structure, which is consistent with the genomic and taxonomic analyses that classify CapO46 as a member of the genus *Rosenblumvirus*. Furthermore, the absence of lysogeny-related and tRNA-encoding genes aligns with the observations of phages in the subfamily *Rakietenvirinae* [39,40]. For phage therapy or biocontrol applications, strictly lytic phages are preferable, as they immediately lyse their host cells. Additionally, since some phages can carry virulence, toxin, or antibiotic resistance genes—thus contributing to the pathogenicity of their bacterial hosts—such characteristics must be avoided [41]. Therefore, the absence of these genes in the CapO46 genome strengthens its genetic safety profile for therapeutic applications.

The host range assay revealed that CapO46 is capable of infecting a wide variety of *S. aureus* isolates from different environmental origins and unrelated countries. The phage isolation host, *S. aureus* O46, is a French isolate obtained from an ewe with mild mastitis, isolated by Vautor et al. (2009) [17] and sequenced by Le Maréchal et al. (2011) [18]. The Brazilian isolates obtained from EMBRAPA dairy cattle have already been evaluated for genetic diversity along with the key virulence factors [20]. Among the isolates analyzed by the authors, those used in our study were clustered into different groups based on a dissimilarity index of 30% using the UPGMA clustering method. Genetic comparisons were also performed between isolates 3059 and UFV2030RH1, revealing significant differences in the categories related to nucleotide, carbohydrate, amino acid, and cofactor metabolism, as well as cell regulation, signaling, and bacteriophages [19]. Furthermore, the antimicrobial susceptibility analysis of these two isolates showed resistance to aztreonam and bacitracin. The isolates obtained from humans (St 10, St 67, St 112, and St 261) are multidrug resistant and have also been genetically analyzed for their similarities [22]. The dendrogram based on PFGE patterns post-digestion with SmaI, associated with the One Health sector and Multidrug Efflux System genes, indicates that isolates St 112 and St 261 were grouped within the same cluster, while St10 and St67 formed a separate cluster. These data indicate considerable genetic differences regarding the Brazilian isolates. As for the Norwegian isolates, we currently lack information on their genotype and antimicrobial susceptibility. Since they were obtained from the same farm, with some originating from the same animal, it is possible that some may be clones. However, considering the geographic distance, substantial environmental differences, and variations in antibiotic therapy between Brazil and Norway [42,43], it is plausible that significant differences also exist between the Brazilian and Norwegian isolates.

Notably, 100% of the *S. aureus* isolates tested were sensitive to the phage, outperforming the previously reported phages in the literature [9,44,45,46,47]. Additionally, high efficiency of plating (EOP > 0.5) was observed in 64.5% of the sensitive isolates. While some isolates exhibited medium or inefficient EOP, this can be attributed to the phage’s exceptionally high efficiency in its isolation host, resulting in elevated titers in that host. In comparison, the relative infection efficiency appears lower in other hosts, but significant phage titers (>10^6^ PFU/mL) were still achieved, even in hosts with inefficient EOP. Moreover, the high EOP observed in almost all the Norwegian isolates may indicate genetic similarities between them, as well as the genetic or environmental factors that render these isolates more susceptible. This raises the possibility of comparative genomic studies between the Brazilian and Norwegian *S. aureus* isolates to identify the genetic determinants of susceptibility.

The kinetic data revealed rapid adsorption, with nearly 100% of the phage particles adsorbed within 10 min, a short latent period of 20 min, and a small burst size of approximately 30 particles per cell. These results are consistent with findings for the phage vB_SauP_phiAGO1.3, another member of the genus *Rosenblumvirus*, which also adsorbs quickly to its host cells, has a short latent period (30 min), and produces a burst size very similar to that of CapO46 (about 35 PFU/cell) [48]. The rapid adsorption and short latent period of CapO46, combined with its ability to easily reach high titers (>10^10^ PFU/mL), underscore its strong replicative potential—a highly desirable trait for phages intended for biocontrol. Moreover, in industrial applications, achieving high phage titers is advantageous as it facilitates both large-scale production and application, optimizing costs and operational efficiency [16,49].

The performance of CapO46 in UHT whole-fat milk contaminated with *S. aureus* demonstrated its effectiveness in reducing bacterial load under practical conditions. The maximum reduction of 7.2 log CFU/mL observed after 12 h highlights the phage’s strong bactericidal effect in a nutrient-rich environment that favors bacterial growth. Even after 48 h of incubation, the phage’s lytic activity persisted, maintaining the host concentration 1.6 logs below that of the control—a 97% reduction. This demonstrates the phage’s strong bactericidal potential, even several hours post-treatment. Previous studies have suggested that heat treatment (UHT > pasteurized) and milk skimming enhances the bactericidal activity of phages by minimizing the inhibitory effects of components found in raw milk [37,50,51]. These raw milk components, including a more complex lipid and protein composition that may inhibit phage binding to host cells, as well as the presence of a microbiota that can slow *S. aureus* growth, could lead to reduced phage propagation in raw milk and, consequently, diminished bactericidal activity. Nevertheless, several studies have already demonstrated the ability of phages to reduce bacterial growth even in raw milk [9,52,53,54].

Evaluating the lytic activity of the CapO46 phage in raw milk will be crucial to further assess its potential for controlling *S. aureus* in more complex contexts, such as those related to bovine mastitis. The phage could be applied, for example, through an intramammary infusion system to treat *S. aureus* infections, as previously demonstrated in preliminary trials using phage-derived endolysins [55]. This approach could not only reduce the costs associated with antibiotic use and the loss of contaminated milk but also lead to improvements in both milk productivity and quality, as prompt mastitis treatment may minimize damage to mammary tissues and enhance the production of healthy milk.

Future studies should also focus on stability assays of the CapO46 phage under different temperature and pH conditions to ensure its viability in varied environments. Additionally, the formulation of phage cocktails, including new phages with diverse specificities, represents a promising perspective to expand the spectrum of action and enhance efficacy in combating *S. aureus*. This approach may also help mitigate the risk of bacterial resistance development, further strengthening the biotechnological potential of phages for industrial and public health applications.

Finally, the results of this study demonstrate that the ability of the phage CapO46 to infect a wide range of *S. aureus* isolates, coupled with its specificity for this species, highlights CapO46 as a promising tool for various applications, particularly in managing infections related to animal health and ensuring food safety in the industry. Its specificity allows for targeted action against the pathogen of interest without affecting beneficial microorganisms, providing a significant biotechnological advantage. Additionally, its ability to reach high titers offers a highly desirable practicality for large-scale application. Lastly, this study provides new insights into the phage’s ability to infect strains from geographically distant, unrelated countries with distinct environmental conditions.

## 5. Conclusions

The results of this study highlight the promising potential of the bacteriophage CapO46 as a biocontrol agent against *S. aureus*. Its high infectivity across multiple isolates, high lytic efficiency, and favorable genomic profile position it as a viable candidate for applications in food safety and animal health. The ability of CapO46 to significantly reduce *S. aureus* populations in UHT whole-fat milk illustrates its lytic efficacy in complex environments. While the phage maintained substantial bactericidal activity for 48 h, the eventual regrowth of *S. aureus* underscores the need for further optimization, such as phage cocktail formulations. Future research should also explore the stability of CapO46 under varying environmental conditions and its performance in more complex contexts, like raw milk. Collectively, these findings support the promotion of phage CapO46 as an innovative and sustainable solution for combating *S. aureus* in critical settings.

## Figures and Tables

**Table 1:** Host range of CapO46 phage and its efficiency of plating (EOP) on different bacterial hosts.

Bacterial Isolate	Source of Isolation	Spot- Test	EOP		
			Average Titer	EOP Value	Production
*Staphylococcus aureus*					
*S. aureus* O46 *	A (Fr)	+		1.0	High
*S. aureus* St 10	H (Br)	+	3.4 × 10^3^	1.68 × 10^−8^	Inefficient
*S. aureus* St 67	H (Br)	+	3.2 × 10^3^	1.62 × 10^−8^	Inefficient
*S. aureus* St 112	H (Br)	+	5.0 × 10^3^	2.5 × 10^−8^	Inefficient
*S. aureus* 222	A (Br)	+	8.7 × 10^9^	1.225	High
*S. aureus* St 261	H (Br)	+	2.0 × 10^5^	8.6 × 10^−6^	Inefficient
*S. aureus* 1334	A (Br)	+	7.6 × 10^7^	0.10	Medium
*S. aureus* UFV2030RH1	A (Br)	+	4.0 × 10^9^	0.53	High
*S. aureus* 3059	A (Br)	+	5.0 × 10^9^	0.791	High
*S. aureus* 3212	A (Br)	+	8.0 × 10^9^	1.076	High
*S. aureus* 3906	A (Br)	+	7.3 × 10^4^	3.6 × 10^−7^	Inefficient
*S. aureus* 3907	A (Br)	+	9.0 × 10^5^	4.5 × 10^−6^	Inefficient
*S. aureus* 4081	A (Br)	+	1.3 × 10^6^	0.0002	Inefficient
*S. aureus* 4182	A (Br)	+	1.1 × 10^10^	1.476	High
*S. aureus* ATCC33591	TC	+	1.0 × 10^6^	5 × 10^−6^	Inefficient
*S. aureus* BO169-1	CM (No)	+	8.0 × 10^9^	0.714	High
*S. aureus* B172-1	CM (No)	+	1.9 × 10^9^	0.169	Medium
*S. aureus* H69Col2	CM (No)	+	1.0 × 10^10^	0.948	High
*S. aureus* H90Col1	CM (No)	+	1.0 × 10^10^	0.951	High
*S. aureus* H90Col2	CM (No)	+	1.3 × 10^10^	1.163	High
*S. aureus* H90Col3	CM (No)	+	1.2 × 10^10^	1.045	High
*S. aureus* H182Col1	CM (No)	+	6.1 × 10^9^	0.547	High
*S. aureus* H249Col1	CM (No)	+	6.1 × 10^9^	0.540	High
*S. aureus* H250Col1	CM (No)	+	7.9 × 10^9^	0.710	High
*S. aureus* H288Col2	CM (No)	+	5.9 × 10^9^	0.532	High
*S. aureus* H297Col1	CM (No)	+	9.9 × 10^9^	0.884	High
*S. aureus* H295Col2	CM (No)	+	6.3 × 10^9^	0.562	High
*S. aureus* H349Col1	CM (No)	+	1.1 × 10^10^	0.944	High
*S. aureus* H350Col1	CM (No)	+	4.1 × 10^9^	0.369	Medium
*S. aureus* H361Col1	CM (No)	+	1.4 × 10^10^	1.281	High
*S. aureus* NCTC8325-4	TC	+	8.3 × 10^9^	0.737	High
Non-aureus staphylococci					
*S. chromogenes* BO226-1	CM (No)	−			
*S. epidermidis* BO5-3	CM (No)	−			
*S. equorum* BO53-1	CM (No)	−			
*S. gallinarum* BO63-3	CM (No)	−			
*S. haemolyticus* BO28-3	CM (No)	−			
*S. sciuri* BO63-2	CM (No)	−			
*S. warneri* BO64-1	CM (No)	−			
*S. xylosus* BO186-3	CM (No)	−			

* CapO46 isolation and propagation host. A (Fr) = Animal (France); A (Br) = Animals (Brazil); H (Br) = Humans (Brazil); CM (No) = Raw cow milk (Norway); TC = Type collections. + = positive for lysis; − = no lysis.

## Data Availability

The original contributions presented in this study are included in the article/Supplementary Materials. Further inquiries can be directed to the corresponding author.

## Supplementary Materials

The following supporting information can be downloaded at: https://www.mdpi.com/article/10.3390/microorganisms13030664/s1, Table S1: *Staphylococcus* spp. isolates used for phage host range investigation and their sources of isolation. The references cited in Table S1 [17,18,19,20,21,22,23] can be found in the Reference list of the main text.

## References

[1] Cheung G.Y.C., Bae J.S., Otto M. (2021). Pathogenicity and Virulence of *Staphylococcus aureus*. Virulence.

[2] Lim K.L., Khor W.C., Ong K.H., Timothy L., Aung K.T. (2023). Occurrence and Patterns of Enterotoxin Genes, Spa Types and Antimicrobial Resistance Patterns in *Staphylococcus aureus* in Food and Food Contact Surfaces in Singapore. Microorganisms.

[3] Léguillier V., Pinamonti D., Chang C.-M., Gunjan, Mukherjee R., Himanshu, Cossetini A., Manzano M., Anba-Mondoloni J., Malet-Villemagne J. (2024). A Review and Meta-Analysis of *Staphylococcus aureus* Prevalence in Foods. Microbe.

[4] Cheng W.N., Han S.G. (2020). Bovine Mastitis: Risk Factors, Therapeutic Strategies, and Alternative Treatments—A Review. Asian-Australas. J. Anim. Sci..

[5] Gonçalves J.L., Kamphuis C., Martins C.M.M.R., Barreiro J.R., Tomazi T., Gameiro A.H., Hogeveen H., dos Santos M.V. (2018). Bovine Subclinical Mastitis Reduces Milk Yield and Economic Return. Livest. Sci..

[6] Melo L.D.R., Oliveira H., Pires D.P., Dabrowska K., Azeredo J. (2020). Phage Therapy Efficacy: A Review of the Last 10 Years of Preclinical Studies. Crit. Rev. Microbiol..

[7] Angelopoulou A., Warda A.K., Hill C., Ross R.P. (2019). Non-Antibiotic Microbial Solutions for Bovine Mastitis—Live Biotherapeutics, Bacteriophage, and Phage Lysins. Crit. Rev. Microbiol..

[8] Breyne K., Honaker R.W., Hobbs Z., Richter M., Żaczek M., Spangler T., Steenbrugge J., Lu R., Kinkhabwala A., Marchon B. (2017). Efficacy and Safety of a Bovine-Associated *Staphylococcus aureus* Phage Cocktail in a Murine Model of Mastitis. Front. Microbiol..

[9] Mohammadian F., Rahmani H.K., Bidarian B., Khoramian B. (2022). Isolation and Evaluation of the Efficacy of Bacteriophages against Multidrug-Resistant (MDR), Methicillin-Resistant (MRSA) and Biofilm-Producing Strains of *Staphylococcus aureus* Recovered from Bovine Mastitis. BMC Vet. Res..

[10] Topka-Bielecka G., Dydecka A., Necel A., Bloch S., Nejman-Faleńczyk B., Węgrzyn G., Węgrzyn A. (2021). Bacteriophage-Derived Depolymerases against Bacterial Biofilm. Antibiotics.

[11] Fernández L., Gutiérrez D., Rodríguez A., García P. (2018). Application of Bacteriophages in the Agro-Food Sector: A Long Way Toward Approval. Front. Cell. Infect. Microbiol..

[12] Siyanbola K.F., Ejiohuo O., Ade-adekunle O.A., Adekunle F.O., Onyeaka H., Furr C.-L.L., Hodges F.E., Carvalho P., Oladipo E.K. (2024). Bacteriophages: Sustainable and Effective Solution for Climate-Resilient Agriculture. Sustain. Microbiol..

[13] Fernández L., Duarte A.C., Rodríguez A., García P. (2021). The Relationship between the Phageome and Human Health: Are Bacteriophages Beneficial or Harmful Microbes?. Benef. Microbes.

[14] Clavijo V., Morales T., Vives-Flores M.J., Reyes Muñoz A. (2022). The Gut Microbiota of Chickens in a Commercial Farm Treated with a *Salmonella* Phage Cocktail. Sci. Rep..

[15] Narayanan K.B., Bhaskar R., Han S.S. (2024). Bacteriophages: Natural Antimicrobial Bioadditives for Food Preservation in Active Packaging. Int. J. Biol. Macromol..

[16] Casey E., Van Sinderen D., Mahony J. (2018). In Vitro Characteristics of Phages to Guide ‘Real Life’ Phage Therapy Suitability. Viruses.

[17] Vautor E., Cockfield J., Le Marechal C., Le Loir Y., Chevalier M., Robinson D.A., Thiery R., Lindsay J. (2009). Difference in Virulence between *Staphylococcus aureus* Isolates Causing Gangrenous Mastitis versus Subclinical Mastitis in a Dairy Sheep Flock. Vet. Res..

[18] Le Maréchal C., Hernandez D., Schrenzel J., Even S., Berkova N., Thiéry R., Vautor E., Fitzgerald J.R., François P., Le Loir Y. (2011). Genome Sequences of Two *Staphylococcus aureus* Ovine Strains That Induce Severe (Strain O11) and Mild (Strain O46) Mastitis. J. Bacteriol..

[19] da Silva Duarte V., Treu L., Sartori C., Dias R.S., da Silva Paes I., Vieira M.S., Santana G.R., Marcondes M.I., Giacomini A., Corich V. (2020). Milk Microbial Composition of Brazilian Dairy Cows Entering the Dry Period and Genomic Comparison between *Staphylococcus aureus* Strains Susceptible to the Bacteriophage VB_SauM-UFV_DC4. Sci. Rep..

[20] Klein R.C., Fabres-Klein M.H., Brito M.A.V.P., Fietto L.G., Ribon A.d.O.B. (2012). *Staphylococcus aureus* of Bovine Origin: Genetic Diversity, Prevalence and the Expression of Adhesin-Encoding Genes. Vet. Microbiol..

[21] Rocha L.S. (2021). Estudo da Variabilidade e Organização de Genes Que Codificam Proteínas de Superfície de Cepas de *Staphylococcus aureus* Associadas à Mastite Bovina.

[22] de Barros M., da Silva Lopes I., Moreira A.J., dos Santos Oliveira Almeida R., Matiuzzi da Costa M., Mota R.A., Nero L.A., Scatamburlo Moreira M.A. (2024). Multidrug Efflux System-Mediated Resistance in *Staphylococcus aureus* under a One Health Approach. World J. Microbiol. Biotechnol..

[23] Bæk K.T., Frees D., Renzoni A., Barras C., Rodriguez N., Manzano C., Kelley W.L. (2013). Genetic Variation in the *Staphylococcus aureus* 8325 Strain Lineage Revealed by Whole-Genome Sequencing. PLoS ONE.

[24] Twest R., Kropinski A.M. (2009). Bacteriophage Enrichment from Water and Soil. Bacteriophages.

[25] Adams M.H. (1959). Bacteriophages.

[26] Sambrook J., Russell D.W. (2001). Molecular Cloning: A Laboratory Manual.

[27] Khan Mirzaei M., Nilsson A.S. (2015). Isolation of Phages for Phage Therapy: A Comparison of Spot Tests and Efficiency of Plating Analyses for Determination of Host Range and Efficacy. PLoS ONE.

[28] Hyman P., Abedon S.T. (2009). Practical Methods for Determining Phage Growth Parameters. Bacteriophages.

[29] Sharifi F., Montaseri M., Yousefi M.H., Shekarforoush S.S., Berizi E., Wagemans J., Vallino M., Hosseinzadeh S. (2024). Isolation and Characterization of Two *Staphylococcus aureus* Lytic Bacteriophages “Huma” and “Simurgh”. Virology.

[30] Nishimura Y., Yoshida T., Kuronishi M., Uehara H., Ogata H., Goto S. (2017). ViPTree: The Viral Proteomic Tree Server. Bioinformatics.

[31] Moraru C., Varsani A., Kropinski A.M. (2020). VIRIDIC—A Novel Tool to Calculate the Intergenomic Similarities of Prokaryote-Infecting Viruses. Viruses.

[32] Turner D., Adriaenssens E.M., Tolstoy I., Kropinski A.M. (2021). Phage Annotation Guide: Guidelines for Assembly and High-Quality Annotation. Phage.

[33] Grant J.R., Enns E., Marinier E., Mandal A., Herman E.K., Chen C., Graham M., Van Domselaar G., Stothard P. (2023). Proksee: In-Depth Characterization and Visualization of Bacterial Genomes. Nucleic Acids Res..

[34] Bortolaia V., Kaas R.S., Ruppe E., Roberts M.C., Schwarz S., Cattoir V., Philippon A., Allesoe R.L., Rebelo A.R., Florensa A.F. (2020). ResFinder 4.0 for Predictions of Phenotypes from Genotypes. J. Antimicrob. Chemother..

[35] Malberg Tetzschner A.M., Johnson J.R., Johnston B.D., Lund O., Scheutz F. (2020). In Silico Genotyping of *Escherichia coli* Isolates for Extraintestinal Virulence Genes by Use of Whole-Genome Sequencing Data. J. Clin. Microbiol..

[36] Joensen K.G., Scheutz F., Lund O., Hasman H., Kaas R.S., Nielsen E.M., Aarestrup F.M. (2014). Real-Time Whole-Genome Sequencing for Routine Typing, Surveillance, and Outbreak Detection of Verotoxigenic *Escherichia coli*. J. Clin. Microbiol..

[37] García P., Madera C., Martínez B., Rodríguez A., Evaristo Suárez J. (2009). Prevalence of Bacteriophages Infecting *Staphylococcus aureus* in Dairy Samples and Their Potential as Biocontrol Agents. J. Dairy. Sci..

[38] Cao S., Yang W., Zhu X., Liu C., Lu J., Si Z., Pei L., Zhang L., Hu W., Li Y. (2022). Isolation and Identification of the Broad-Spectrum High-Efficiency Phage VB_SalP_LDW16 and Its Therapeutic Application in Chickens. BMC Vet. Res..

[39] Hua Y., An X., Pei G., Li S., Wang W., Xu X., Fan H., Huang Y., Zhang Z., Mi Z. (2014). Characterization of the Morphology and Genome of an *Escherichia coli* Podovirus. Arch. Virol..

[40] Park S.Y., Kwon H., Kim S.G., Park S.C., Kim J.H., Seo S. (2023). Characterization of Two Lytic Bacteriophages, Infecting *Streptococcus bovis*/*equinus* Complex (SBSEC) from Korean Ruminant. Sci. Rep..

[41] Jiang T., Guo C., Wang M., Wang M., Zhang X., Liu Y., Liang Y., Jiang Y., He H., Shao H. (2020). Genome Analysis of Two Novel *Synechococcus* Phages That Lack Common Auxiliary Metabolic Genes: Possible Reasons and Ecological Insights by Comparative Analysis of Cyanomyoviruses. Viruses.

[42] Rajala-Schultz P., Nødtvedt A., Halasa T., Persson Waller K. (2021). Prudent Use of Antibiotics in Dairy Cows: The Nordic Approach to Udder Health. Front. Vet. Sci..

[43] Andretta M., Call D.R., Nero L.A. (2023). Insights into Antibiotic Use in Brazilian Dairy Production. Int. J. Dairy. Technol..

[44] Wang Z., Zheng P., Ji W., Fu Q., Wang H., Yan Y., Sun J. (2016). SLPW: A Virulent Bacteriophage Targeting Methicillin-Resistant *Staphylococcus aureus* In Vitro and In Vivo. Front. Microbiol..

[45] Ji J., Liu Q., Wang R., Luo T., Guo X., Xu M., Yin Q., Wang X., Zhou M., Li M. (2020). Identification of a Novel Phage Targeting Methicillin-Resistant *Staphylococcus aureus* In Vitro and In Vivo. Microb. Pathog..

[46] Titze I., Lehnherr T., Lehnherr H., Krömker V. (2020). Efficacy of Bacteriophages Against *Staphylococcus aureus* Isolates from Bovine Mastitis. Pharmaceuticals.

[47] Kolenda C., Medina M., Bonhomme M., Laumay F., Roussel-Gaillard T., Martins-Simoes P., Tristan A., Pirot F., Ferry T., Laurent F. (2022). Phage Therapy against *Staphylococcus aureus*: Selection and Optimization of Production Protocols of Novel Broad-Spectrum *Silviavirus* Phages. Pharmaceutics.

[48] Głowacka-Rutkowska A., Gozdek A., Empel J., Gawor J., Żuchniewicz K., Kozińska A., Dębski J., Gromadka R., Łobocka M. (2019). The Ability of Lytic Staphylococcal Podovirus VB_SauP_phiAGO1.3 to Coexist in Equilibrium with Its Host Facilitates the Selection of Host Mutants of Attenuated Virulence but Does Not Preclude the Phage Antistaphylococcal Activity in a Nematode Infection Model. Front. Microbiol..

[49] Gill J., Hyman P. (2010). Phage Choice, Isolation, and Preparation for Phage Therapy. Curr. Pharm. Biotechnol..

[50] O’Flaherty S., Coffey A., Meaney W.J., Fitzgerald G.F., Ross R.P. (2005). Inhibition of Bacteriophage K Proliferation on *Staphylococcus aureus* in Raw Bovine Milk. Lett. Appl. Microbiol..

[51] Gill J.J., Sabour P.M., Leslie K.E., Griffiths M.W. (2006). Bovine Whey Proteins Inhibit the Interaction of *Staphylococcus aureus* and Bacteriophage K. J. Appl. Microbiol..

[52] Son H.M., Duc H.M. (2024). Prevalence and Phage-Based Biocontrol of Methicillin-Resistant *Staphylococcus aureus* Isolated from Raw Milk of Cows with Subclinical Mastitis in Vietnam. Antibiotics.

[53] Badiyal A., Dhial K., Singh G., Dhar P., Sharma M., Verma S. (2024). Isolation, Characterization and In Vitro Evaluation of Novel Lytic Phages Active Against *Staphylococcus aureus* and *Escherichia coli* of Bovine Mastitis Origin. Proc. Natl. Acad. Sci. India Sect. B Biol. Sci..

[54] McLean S.K., Dunn L.A., Palombo E.A. (2013). Phage Inhibition of *Escherichia Coli* in Ultrahigh-Temperature-Treated and Raw Milk. Foodborne Pathog. Dis..

[55] Fan J., Zeng Z., Mai K., Yang Y., Feng J., Bai Y., Sun B., Xie Q., Tong Y., Ma J. (2016). Preliminary Treatment of Bovine Mastitis Caused by *Staphylococcus aureus*, with Trx-SA1, Recombinant Endolysin of *S. aureus* Bacteriophage IME-SA1. Vet. Microbiol..

