# Supplementary material for: Characterization of Newly Isolated Rosenblumvirus Phage Infecting Staphylococcus aureus from Different Sources
Source: Microorganisms. 2025 Mar 15;13(3):664. doi: 10.3390/microorganisms13030664 (PMC11945092; doi:10.3390/microorganisms13030664)
Supplement: Supplementary file 1 [file microorganisms-13-00664-s001.zip › microorganisms-3513900-supplementary.pdf]

Supplementary Materials

Table S1. *Staphylococcus* spp. isolates used for phage host range investigation and their sources of isolation.

Source	Isolate	Source description	Sampling location	Reference
Animal (France)	*S. aureus* O46	Ewe with mild mastitis, from a dairy sheep farm	Southeast of France	[17,18]
Animals (Brazil)	*S. aureus* 222	-	-	-
	S. aureus 1334	-	-	-
	S. aureus 3059 *	Dairy cow with clinical mastitis	Brazil	[19]
	S. aureus 3906 *	Dairy cow with subclinical mastitis	Rio de Janeiro, RJ, Brazil	[20]
	S. aureus 3907 *	Dairy cow with subclinical mast19itis	Rio de Janeiro, RJ, Brazil	[20]
	S. aureus 3212 *	Dairy cow with subclinical mastitis	Rio de Janeiro, RJ, Brazil	[20]
	S. aureus 4081 *	Dairy cow with subclinical mastitis	Juíz de Fora, MG, Brazil	[20]
	S. aureus 4182 *	Dairy cow with subclinical mastitis	Patrocínio, MG, Brazil	[21]
	S. aureus UFV2030RH1	Raw cow milk of a dairy cow at drying-off period	Viçosa, MG, Brazil	[19]
Humans (Brazil)	*S. aureus* St 10	Orthopedic screw	-	[22]
	S. aureus St 67	Blood culture	-	[22]
	S. aureus St 112	Tracheal secretion	-	[22]
	S. aureus St 261	Skull bone	-	[22]
Type collections	*S. aureus* ATCC 33591	-	-	-
	S. aureus NCTC 8325-4 **	-	-	[23]
Raw cow milk (Norway)	*S. aureus* BO169-1	Isolated 2018, Farm 2	Ås, Norway	-
	S. aureus B172-1	Isolated 2018, Farm 1	Ås, Norway	-
	S. aureus H69Col2	Farm 1, Cow1, isolated on 15/11/2022	Ås, Norway	-

S. aureus H90Col1	Farm 1, Cow2, isolated 15/11/2022	Ås, Norway	-
S. aureus H90Col2	Farm 1, Cow2, isolated 15/11/2022	Ås, Norway	-
S. aureus H90Col3	Farm 1, Cow2, isolated 15/11/2022	Ås, Norway	-
S. aureus H182Col1	Farm 1, Cow2, isolated 16/02/2023	Ås, Norway	-
S. aureus H249Col1	Farm 1, Cow1, isolated 09/05/2023	Ås, Norway	-
S. aureus H250Col1	Farm 1, Cow1, isolated 09/05/2023	Ås, Norway	-
S. aureus H288Col2	Farm 1, Cow2, isolated 30/05/2023	Ås, Norway	-
S. aureus H297Col1	Farm 1, Cow3, isolated 08/06/2023	Ås, Norway	-
S. aureus H295Col2	Farm 1, Cow4, isolated 08/06/2023	Ås, Norway	-
S. aureus H349Col1	Farm 1, Cow1, isolated 29/08/2023	Ås, Norway	-
S. aureus H350Col1	Farm 1, Cow1, isolated 29/08/2023	Ås, Norway	-
S. aureus H361Col1	Farm 1, Cow5, isolated 29/08/2023	Ås, Norway	-
S. chromogenes BO226-1	-	Ås, Norway	-
S. epidermidis BO5-3	-	Ås, Norway	-
S. equorum BO53-1	-	Ås, Norway	-
S. gallinarum BO63-3	-	Ås, Norway	-
S. haemolyticus BO28-3	-	Ås, Norway	-
S. sciuri BO63-2	-	Ås, Norway	-
S. warneri BO64-1	-	Ås, Norway	-
S. xylosus BO186-3	-	Ås, Norway	-

* Kindly provided by EMBRAPA Dairy Cattle (Juiz de Fora, MG, Brazil).

** Kindly provided by Professor Morten Kjos, NMBU (Norway).
